# Performance Evaluation of a Quality of Service Control Scheme in Multi-Hop WBAN Based on IEEE 802.15.6

**DOI:** 10.3390/s18113969

**Published:** 2018-11-15

**Authors:** Kento Takabayashi, Hirokazu Tanaka, Chika Sugimoto, Katsumi Sakakibara, Ryuji Kohno

**Affiliations:** 1 Department of Information and Communication Engineering, Faculty of Computer Science and Systems Engineering, Okayama Prefectural University, 111 Kuboki, Soja, Okayama 719-1197, Japan; sakaki@c.oka-pu.ac.jp; 2 Graduate School of Information Sciences, Hiroshima City University, 3-4-1, Ozuka-Higashi, Asa-Minami-Ku, Hiroshima 731-3194, Japan; hi.tanaka@m.ieice.org; 3 Faculty of Engineering, Yokohama National University, 79-5 Tokiwadai, Hodogaya-Ku, Yokohama 240-8501, Japan; sugimoto-chika-zb@ynu.ac.jp (C.S.); kohno-ryuji-ns@ynu.ac.jp (R.K.)

**Keywords:** wireless body area networks, multi-hop, IEEE802.15.6, error controlling, communication distance, QoS

## Abstract

The performance of a quality of service (QoS) control scheme in a multi-hop wireless body area network (WBAN) based on the IEEE Std. 802.15.6 is evaluated. In medical Internet of Things systems, WBANs are an important technology. In a previous study, an optimal quality of service control scheme that employs a multiplexing layer for priority scheduling and a decomposable error control coding scheme for WBANs were proposed. However, the two-hop extension supported by IEEE Std.802.15.6 has not been considered. Here, the two-hop extension is applied. Then, the packet error ratio, number of transmissions, and energy efficiency of our previously proposed system are compared to a standard scheme under several conditions. Also, novel evaluations based on communication distance are conducted. Numerical results demonstrate that our proposed scheme, in which coding rates change relative to channel conditions, outperforms standard schemes in many aspects. In addition, those systems show the best performance when the communication distance of the first hop equals that of the second hop. In addition, the above result is theoretically clarified.

## 1. Introduction

Health monitoring systems that employ wearable vital sign sensors and wireless communication (referred to as medical Internet of Things (m-IoT) systems) have received significant attention recently [1,2,3,4,5,6,7,8,9,10,11,12,13,14,15,16]. Wireless body area networks (WBAN) are an important key technology in the m-IoT field. WBAN sensors can sample, monitor, process, and communicate a significant amount of various vital data [14]. In addition, they can provide real-time feedback [14]. It is expected that WBANs will be implemented to monitor patient health. In particular, they are expected to monitor elderly people in hospitals, nursing homes, and their own homes [15].

Recently, there have been various attempts to develop standards for WBAN systems [17,18]. In 2011, IEEE Std. 802.15.4a was issued as a standard for wireless personal area networks (WPAN) assuming a wide range of applications [19]. However, IEEE Std. 802.15.4a has not been optimized for medical and healthcare applications. Therefore, IEEE Std. 802.15.6 was published as a standard specialized mainly for implant and wearable WBAN assuming medical-healthcare uses (but not limited to them). In WBAN systems, a wearable vital sign sensor node can include various types of sensors with different data rates. In addition, the allowable communication error ratio and delay depend on the application. IEEE Std. 802.15.6 aims to provide an international standard for low power, short range, and extremely dependable wireless communication within the surrounding area of the human body, supporting a vast range of data rates [14]. Additionally, IEEE Std. 802.15.6 defines eight user priority levels. Quality of Service (QoS) control must ensure that different data types are communicated effectively and efficiently. Therefore, optimal QoS control for input data is an important feature in sensor data transmission procedures. To address this requirement, an optimal QoS control scheme that employs a multiplexing layer for priority scheduling and a decomposable error control coding scheme that adapts to varying channel conditions or QoS requirements have been proposed [20,21,22]. Here, the target WBAN consists of a wearable sensor device that includes multiple sensors whose output data are transmitted using a common medium access control (MAC) and the physical layer (PHY). In those studies, simulations and a theoretical analysis were performed to evaluate the performance of the proposed system by comparing it to an IEEE Std.802.15.6-based system [20,21,22].

Note that IEEE Std. 802.15.6 supports a two-hop extension. In this study, the performance of an error control scheme for a multi-hop WBAN based on IEEE Std. 802.15.6 is evaluated. Specifically, the packet delivery failure ratio (PDFR), number of transmissions as a replacement for delay, and energy efficiency which is throughput considering energy consumption of our previously proposed error control scheme and IEEE Std. 802.15.6 are evaluated relative to this standard’s two-hop extension [20,21,22] like under multi-path fading channel of ultra-wideband (UWB) PHY. The main contribution and novelty of the manuscript are as follows:

1. The performance of our previously proposed QoS control scheme is improved by appropriately determining the coding rate using channel estimation. With this improvement, data packets can be relayed to the hub with a small number of transmissions even when the maximum number of retransmissions is limited by a two-hop extension.
2. Novel performance evaluations are conducted as a function of the distance between transmitter and receiver assuming a real environment, which were not considered in our previous work [20,21,22]. Through these evaluations, we confirm that our proposed QoS control method is effective even assuming a real environment. It is also clear that it is better to perform two-hop expansion than one-hop case by setting the distance between transmitter and receiver in each hop appropriately. In addition, this paper contributes to theoretically clarifying the relevant optimum setting.

The remainder of this paper is organized as follows. Section 2 introduces the related research in the field of this manuscript. In Section 3, the related descriptions of IEEE Std. 802.15.6 are explained. Section 4 shows our previously proposed error control method. The system model is described in Section 5. Computer simulated performance evaluations and theoretical analysis of the results are presented in Section 6. Section 7 concludes the paper.

## 2. Related State of the Art Research

This section introduces the latest research related to this manuscript.

WBANs extended to multi-hop communication have been studied to increase their lifetime. Many studies on multi-hop WBANs have focused on energy-efficient MAC or routing protocols [23,24,25,26,27,28,29,30]. For example, previous studies have focused on a cross-layer technique that includes a MAC layer to reduce delay and improve energy efficiency [23,24,25]. In [23], a network tree in a distributed manner has been used to guarantee collision free access to the medium and to route data towards the sink. Computer simulation results have shown that the protocol offers low delay and good resilience to mobility. The proposed solution of [24] extended the cooperation at the MAC layer to a cross-layered gradient based routing solution that allows interaction between WBAN and environmental sensors to ensure data delivery from WBANs to a distant gateway. The MAC layer of [25] provided the network layer with local information about the quality of on-body links to enable the WBAN to identify the most reliable links in a distributed manner. Results of [25] have shown the effectiveness of the proposed design which takes advantage of dynamic scheduling and multi-hop relays as warranted by the link conditions. An energy-efficient and low-delay relay selection method for multi-hop WBANs has also been discussed [26,27]. In [26], a game-theory approach has been proposed to investigate the problem of relay selection and power control with QoS constraints in multiple-access WBANs. Reference [27] considered adaptive power control and routing in multi-hop WBANs, and developed a low overhead energy-efficient routing scheme. The proposed routing protocol has established an energy-efficient end-to-end path as well as adaptively choosing transmission power for sensor nodes. Path loss models have also been considered to evaluate the energy efficiency of multi-hop WBAN topologies in [28]. Ref. [28] has discussed the propagation channel between two half-wavelength dipoles at 2.45 GHz placed near a human body, and then presented an application for cross-layer design to optimize the energy consumption of different topologies. In addition, a security scheme based on PHY characteristics for multi-hop WBANs has been described [29,30]. The game-theory framework of [29] was proposed, wherein wearable sensor devices interact in the presence of wiretappers and under fading channel conditions to find the most secure multi-hop path to the hub, while adhering to the end-to-end delay requirements. Reference [30] proposed MASK-BAN, a lightweight fast authenticated secret key extraction scheme for intra-WBAN communication. However, those studies did not focus on an error control scheme. 

On the other hand, the analytical expressions for energy efficiency and packet error ratio (PER) have been formulated for two-way relay cooperative communication in [31] which is similar to our work. Then, [31] introduced a hybrid system, which allows switching between the proposed two-way relay, multi-stage one-way relay, and direct link to maximize energy efficiency, and a joint network-channel coding scheme using convolutional and BCH codes. However, [31] did not consider a hybrid automatic repeat request (ARQ), which was applied to our method. In addition, path loss, shadowing, and additive white Gaussian noise (AWGN) have been taken into account, but multi-path or flat fading has not in [31]. On the other hand, this manuscript considers all of them.

## 3. Related Description of IEEE Std. 802.15.6

### 3.1. UWB PHY

The IEEE Std. 802.15.6 defines three PHY layers: narrowband (NB), UWB, and human body communications (HBC). This study focused on an impulse radio ultra-wideband PHY layer (IR-UWB-PHY) which offers high data rate transmission, low energy consumption, powerful multi-pass resolution, good coexistence with other wireless communication systems, and so on.

**Figure 1:**
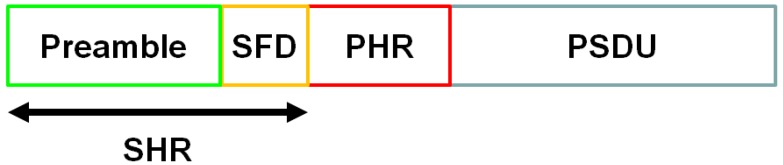
Ultra-wideband physical layer frame format. SFD, SHR, PHR, and PSUD are abbreviations of start-of-frame delimiter, synchronization header, physical layer header, and physical layer service data unit respectively.

The UWB PHY frame format is formed by the synchronization header (SHR), the physical layer header (PHR), and the physical layer service data unit. (PSDU), respectively, as shown in Figure 1 [17]. The PSDU contains the MAC protocol data unit (MPDU) and the BCH parity bits. The PSDU can therefore be regarded as the payload. The information contained by the PHR includes the data rate of the PSDU and the length of the MAC frame body, and the SHR contains the preamble used for timing synchronization, packet detection, and other purposes, and the start-of-frame delimiter (SFD) for frame synchronization. This research mainly focused on the performance of the payload (PSDU).

### 3.2. Two-Hop Extension

**Figure 2:**
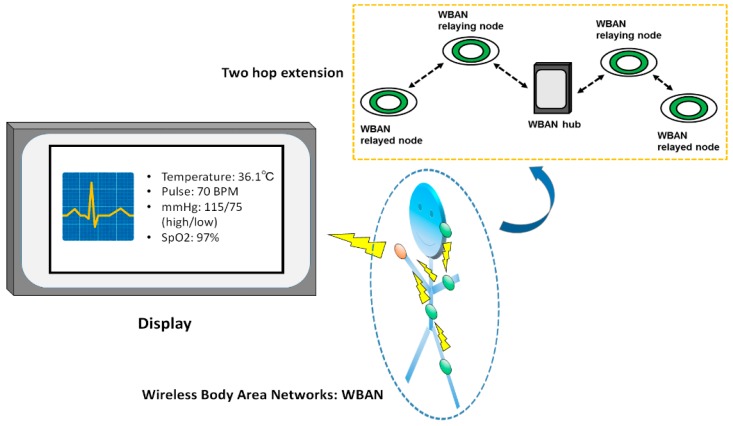
Example smart health care monitoring system.

An example smart health care monitoring system that includes a WBAN with a two-hop extended star network topology is shown in Figure 2. As can be seen, vital information obtained by WBAN nodes is displayed on a monitoring unit through a WBAN hub.

In IEEE Std. 802.15.6, a node and a hub can utilize the two-hop extension to exchange frames through another node, except in the medical implant communication service band. In Figure 2, the terminal, intermediate nodes, and the hub function as relayed nodes, relaying nodes, and the target hub of a relayed node, respectively. Here, a relayed node or the target hub can initiate a two-hop extension at times determined by the initiator. Note that a relaying node can exchange its frames with the hub directly.

A relayed node shall not send its frames to a relaying node in contended allocations provided by the target hub [17]. Thus, a scheduled access phase can only be utilized in the case of a two-hop extension. Therefore, the managed access phase as defined in IEEE Std. 802.15.6 is only used in this study.

## 4. Previously Proposed Error Control Method

**Figure 3:**
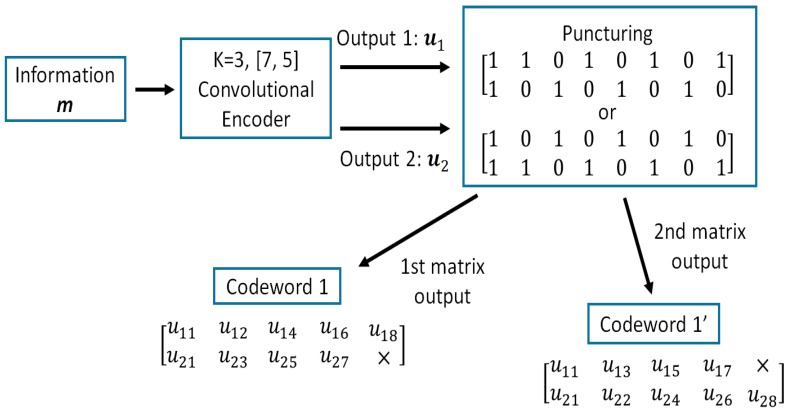
Punctured convolutional codes $r_{c}=8/9$.

In a previous study, an optimal QoS control scheme that employs decomposable error control coding and Weldon’s ARQ scheme was proposed [20,21,22]. As an example of the decomposable code, punctured convolutional code (constraint length K=3; coding rates $r_{c}$ are 8/9 to 1/16) is used. The $r_{c}=8/9$ punctured code patterns (codeword 1 and codeword 1’) are generated from a convolutional code whose generator polynomial is [7,5] and the coding rate is $r_{c}$ = 1/2 as shown in Figure 3.

**Figure 4:**
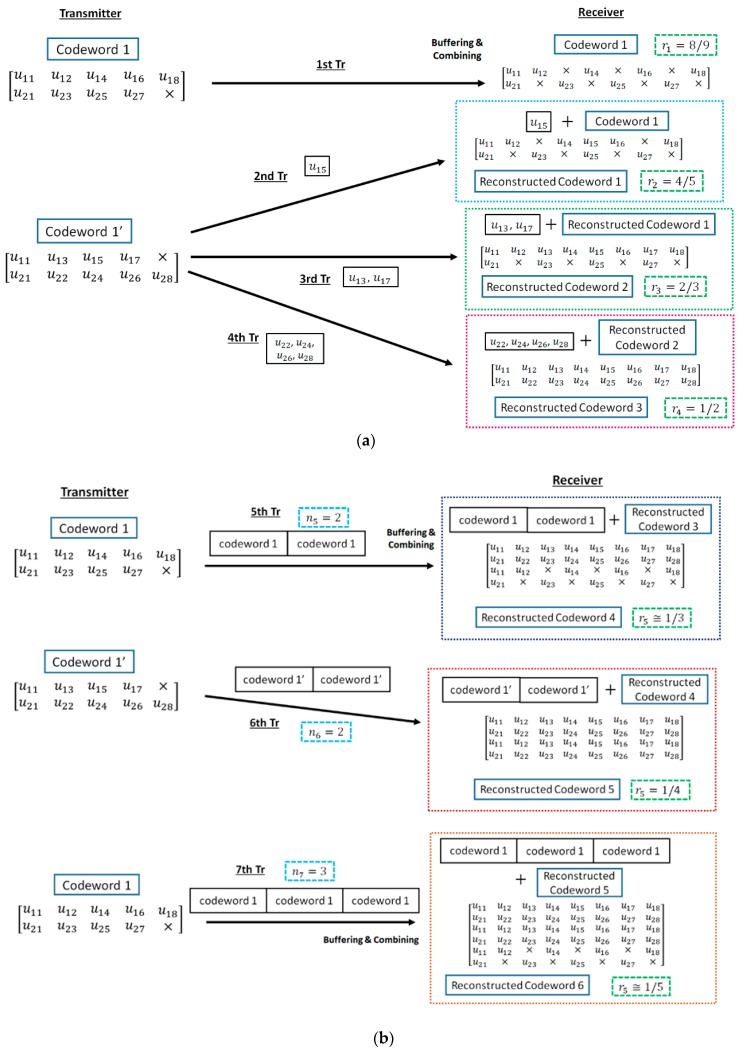
Method of reconstructing decomposable codes. (**a**) $1/2\leq r_{i}\leq 8/9;$ (**b**) $r_{i}\leq 1/2$ [20,21,22].

In our proposed method, retransmission is performed as follows (Figure 4) [20,21,22]:

1. Firstly, the information bit sequence m is encoded via the punctured convolutional code, and codeword 1 is transmitted.
2. If bit errors are detected after decoding codeword 1, the receiver stores the transmitted codeword 1, and the transmitter re-sends the sub-codeword of codeword 1′ $n_{i}$ times if 1 ≤i ≤3. At the receiver, the received sub-codeword and stored codeword are combined, and the reconstructed codeword is decoded.
3. After the third retransmission, codeword 1 is sent $n_{4}$ times and combined with a buffered codeword at the receiver. If bit errors are detected after decoding reconstructed codeword, the $n_{4}\;$codeword 1 is buffered in the receiver, and codeword 1′ is transmitted $n_{5}$ times and combined with a stored codeword.
4. After that, codeword 1 and 1′ are sent alternately $n_{i}$ times and stored. Then, a receiver reconstructs and decodes low-rate decomposable codes by changing the number of data copies $n_{i}$ in Weldon’s ARQ protocol. At this time, a buffered old codeword is updated to a transmitted new codeword.
5. This operation continues until no bit errors are detected or the maximum number of transmissions q is achieved.

**Figure 5:**
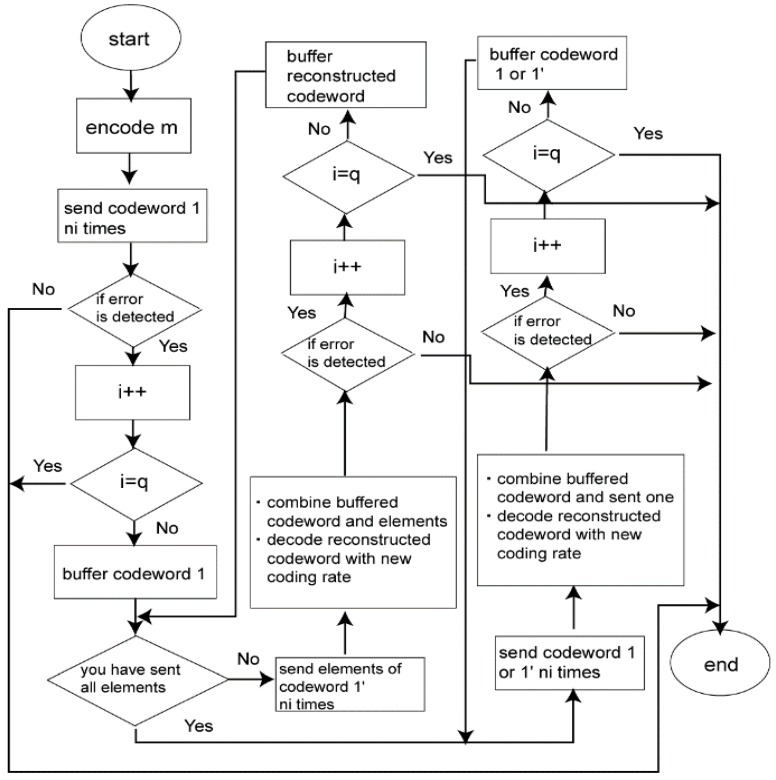
Flowchart of the proposed automatic repeat request (ARQ) protocol [20,21,22].

Figure 5 shows a flowchart of the protocol of our proposed error correcting scheme.

This scheme has the following advantages [21,22]. The first one is that the coding rate is very wide. Hence, bit (or packet) errors can be sufficiently eliminated by the coding rate of $r_{c}$ = 8/9 under very good channel conditions, while very low coding rates can remove bit errors under bad channel conditions. As for the second advantage, in the case of a small number of retransmissions, it is sufficient to transmit a small number of redundant bits. This characteristic leads to improvement of energy efficiency and reduction of transmission delay on retransmission. Finally, combining characteristics of Weldon’s ARQ protocol makes it possible to perform wider QoS control. That is, by controlling the number of data copies $n_{i}$ in Weldon’s ARQ protocol for transmission, the error correcting capability can be changed even if an error correcting code with the same coding rate is used.

## 5. System Model in Two-Hop Case

### 5.1. System Model and QoS Requirement

**Figure 6:**
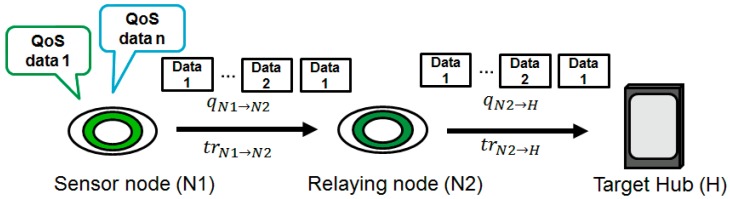
System model.

It is assumed that a sensor node (*N*1) includes multiple sensors that produce different data types that are transmitted via a relaying node (*N*2) to the target hub (*H*) (Figure 6). Here, $tr_{A\to B}$ is the number of transmissions from nodes A to B and $q_{A\to B}$ is the maximum number of transmissions from nodes A to B. If bit errors are detected, the system retransmits until the maximum number of retransmissions is reached. Then, the transmission is considered to have failed if the data from a sensor node do not reach the target hub.

The average number of transmissions from node A to node B $\bar{tr_{A\to B}}$ is expressed as follows:

(1) $$\;\bar{tr_{A\to B}}=\overset{q_{A\to B}-1}{\underset{i=1}{\sum }}i\overset{i}{\underset{j=1}{\prod }}P_{fail,j-1}\left(1-P_{fail,i}\right)+q_{A\to B}\overset{q_{A\to B}}{\underset{j=1}{\prod }}P_{fail,j-1}\;(*\;P_{fail,0}=1).$$

Here, $P_{fail,i}$ is the probability of transmission failure in the ith transmission. In this study, $P_{fail,i}$ [21] is the same as PER because packet collisions in the MAC layer are not considered. Hence, bit errors are taken into account due to noise and multipath fading. Then, the average number of transmissions in a two-hop case is expressed as follows:

(2) $$\;\bar{tr_{2hop}}=\{\begin{array}{c} q_{N1\to N2}\;\left(P_{fail,1st}=1\right)\\ \bar{tr_{N1\to N2}}+\bar{tr_{N2\to H}}\;\left(P_{fail,1st}\neq 1\right) \end{array}\;(*\;\sum_{i=1}^{-1}f\left(i\right)=0,\;\sum_{i=1}^{0}f\left(i\right)=0).$$

Here, $P_{fail,1st}$ is the probability of transmission failure at the first hop.

In this study, two data (Data A and Data B) with different types of QoS requirements are considered. Here, it is assumed that a low PER is desired for Data A and high energy efficiency is important for Data B as an example [20,21,22]. As the first reason for selecting those QoSs, this paper particularly focused on an error controlling scheme utilizing a hybrid ARQ, and then PER and the energy efficiency are very important parameters for evaluation of such a scheme. Secondly, those two parameters are related to a trade-off. Hence, we also aimed to show the relationship in some evaluations. Data A is assumed to be a physiological parameter with a low data rate, for example blood pressure, SpO2, or temperature, and Data B to be a waveform, such as an ECG output [20,21,22]. The transmission order and error control process of different types of data packets depend on such QoS requirements. The characteristics of different data types are summarized in Table 1 [13,14,22].

**Figure 7:**
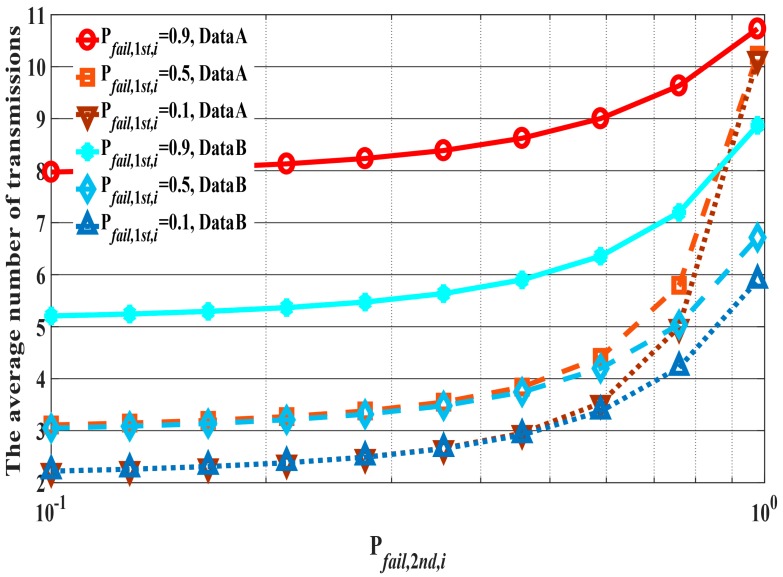
Examples of $\bar{tr_{2hop}}$. $P_{fail,1st,i}=0.9,\;\;0.5,$ and 0.1. $P_{fail,2nd,i}$ is in the range 0.1–0.9.

Then, each $q_{A\to B}$ is set as shown in Table 2. The maximum number of retransmissions is four in high QoS mode in the IR-UWB (impulse radio ultra-wideband) PHY of the standard. However, the default mode in the IR-UWB PHY, the narrowband PHY, and the Human Body Communication PHY in IEEE Std. 802.15.6 do not define a maximum number of retransmissions. Thus, in the current study, this parameter was set according to the QoS requirements of the data in our previous work [20,21,22]. Figure 7 shows examples of each $\bar{tr_{2hop}}$. $P_{fail,1st,i}$ and $P_{fail,2nd,i}$ denote the probability of transmission failure at the ith transmission of the first and second hop, respectively. With Data A, $\bar{tr_{2hop}}$ increases steeply under high $P_{fail,2nd,i}$ conditions, especially in low $P_{fail,1st,i}$ cases, because $tr_{N2\to H}$ increases towards $q_{max}$ as $P_{fail,2nd,i}$ increases. On the other hand, $\bar{tr_{2hop}}$ increases gradually with Data B because $q_{N1\to N2}$ and $q_{N2\to H}$ are constant.

### 5.2. Two Proposed Schemes

**Figure 8:**
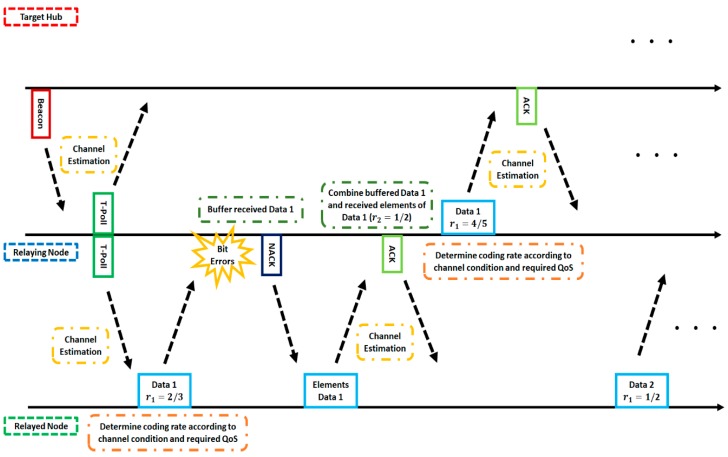
Operation example of Scheme 2. ACK, NACK, and QoS are abbreviations of acknowledgement, negative-acknowledgement, and quality of service respectively.

In this study, two proposed schemes are assumed. The first scheme (Scheme 1) transmits data depending on preset parameters, which was used in our previous study [20,21,22]. On the other hand, in the second scheme (Scheme 2), coding rates are varied with the SNR estimated using a preamble signal according to each QoS requirement (e.g., desired bit error ratio (BER)), which is introduced for the first time in this manuscript. The operation example is shown in Figure 8. Firstly, the channel SNR is estimated by using the preamble of the beacon or the T-Poll received from the hub or the relaying node. Next, the relayed node or the relaying node determines the coding rate according to the estimated channel SNR and transmits data to the relaying node or the hub. If a bit error is detected, elements of the encoded data (codeword) are transmitted to increase error correcting capability after receiving negative-acknowledgement (NACK). Then, if data are transmitted successfully, the channel SNR is estimated by using the returned acknowledgement (ACK) preamble, the coding rate is determined, and the next data are sent. Since Scheme 2 uses an existing preamble, extra overhead is not required.

Then, the channel SNR is estimated using the following equations [32]:

(3) $$\;\Gamma =\frac{{\left|\rho \right|}^{2}}{1-{\left|\rho \right|}^{2}}\;$$

(4) $$\;\rho =\frac{x^{H}r}{\sqrt{x^{H}x}\sqrt{r^{H}r}}\;$$

(5)  0≤ρ≤1 

(6)  x=s+η 

(7)  r=cs 

Here, Γ is the estimated SNR, ρ is a correlation coefficient, x is a preamble signal with noise η, and r is a preamble signal that consists of a signal s and unknown constant *c* without noise or interference. Then, we explain why Equation (3) becomes the SNR. Let’s substitute Equation (4) into Equation (3):

(8) $$\;\Gamma =\frac{{\left|x^{H}r\right|}^{2}}{x^{H}xr^{H}r-{\left|x^{H}r\right|}^{2}}\;.\;$$

Here, ${\left|x^{H}r\right|}^{2}$ can be expanded as follows:

(9) $$\;{\left|x^{H}r\right|}^{2}={\left|s^{H}r+\eta^{H}r\right|}^{2}=\left(s^{H}r+\eta^{H}r\right){\left(s^{H}r+\eta^{H}r\right)}^{*}=\left(s^{H}r+\eta^{H}r\right)\left(r^{H}s+r^{H}\eta \right)=s^{H}rr^{H}s\;.\;$$

Here, $\eta^{H}r=r^{H}\eta =0$ since noise and a preamble signal are uncorrelated. Then, $x^{H}xr^{H}r$ can be expanded as follows:

(10) $$x^{H}xr^{H}r=s^{H}sr^{H}r+\eta^{H}\eta r^{H}r.$$

For the same reason, $\eta^{H}s=s^{H}\eta =0$. Then, Equation (8) can be modified from Equations (9) and (10) as follows:

(11) $$\;\Gamma =\frac{s^{H}rr^{H}s}{s^{H}sr^{H}r+\eta^{H}\eta r^{H}r-s^{H}rr^{H}s}\;.\;$$

Additionally, $s^{H}sr^{H}r=s^{H}rr^{H}s$ as follows:

(12) $$s^{H}rr^{H}s=\;s^{H}\left(cs\right)\left(cs^{H}\right)s=\;s^{H}s\left(cs^{H}\right)\left(cs\right)=s^{H}sr^{H}r.$$

Finally, Equation (11) is summarized as follows, indicating that the SNR can be derived:

(13) $$\;\Gamma =\frac{s^{H}sr^{H}r}{\eta^{H}\eta r^{H}r}=\frac{s^{H}s}{\eta^{H}\eta }=\frac{P_{s}}{P_{\eta }}\;$$

where $P_{s}$ and $P_{\eta }$ are signal power and noise power, respectively.

**Figure 9:**
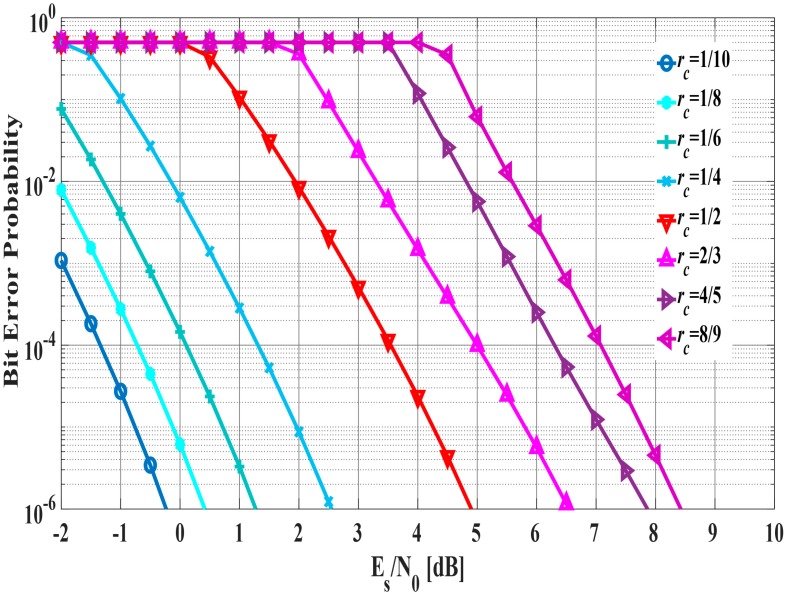
Bit error probability in each coding rate.

In Scheme 2, the criteria to determine the coding rate are expressed as follows:

(14) $$\;\mathrm{Desired}\;\mathrm{BER}=1-{\left(1-\mathrm{Disered}\;\mathrm{PER}\right)}^{\frac{1}{L_{info}}}\;$$

where $L_{info}$ is length of information bits. Hence, the desired BER is calculated from the desired PER such as Table 1 and $L_{info}$. The coding rate is determined based on that and the estimated SNR from Figure 9. As the reason for using the desired PER and $L_{info}$, it is possible to accurately obtain the desired BER for determining the coding rate from the Equation (14) since the required QoS (desired PER) is used. For example, in a case where the desired PER is $10^{-2}$ and $L_{info}$ is 400 bits, the desired BER is calculated as $2.5\times 10^{-5}$. Here, if the estimated SNR is 5 dB, the coding rate is determined to be $r_{c}$ = 1/2 as shown in Figure 9.

## 6. Results and Discussion

### 6.1. Performance Evaluation by Computer Simulation

In this section, the proposed and standard schemes with two-hop extension are evaluated based on communication distance by computer simulations. The computer simulator was built by us with MATLAB. The main simulation parameters are listed in Table 3 and refer to our previous work [20,21,22]. Table 4 shows the preset parameters of Weldon’s ARQ protocol at the ith transmission for Scheme 1 [20,21,22]. The computer simulation assumes that there is no error in SHR and PHR. That is, only the characteristics of PSDU are evaluated. In computer simulations of the compared schemes, Data A was transmitted using the default mode with (63, 51) BCH code in IEEE Std. 802.15.6 and the error control scheme utilizing the (63, 55) Reed-Solomon code in IEEE Std. 802.15.4a with ordinary ARQ, whereas Data B was transmitted using the high QoS mode with (126, 63) shortened BCH code and type-II hybrid ARQ, and then the error control scheme utilizing the concatenated code consisting of the (63, 55) Reed–Solomon code and the convolutional code whose constraint length is three and coding rate is 1/2 in IEEE Std. 802.15.4a with ordinary ARQ [17,19]. In these computer simulations, the IEEE model CM 3 is applied as a channel model, which is targeted for wearable WBAN and includes multi-path fading [33]. Then, a hospital room case in the IEEE model CM3 is utilized as a path loss model [33]. The path loss is expressed as follows:

(15) $$\;PL\left(d\right)=a\times log_{10}d+b+N\;$$

**Figure 10:**
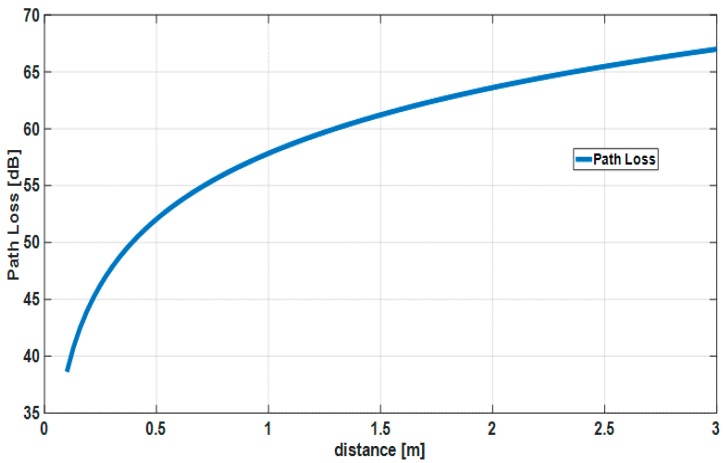
Average path loss in IEEE model CM3.

Here, a and b are linear fitting coefficients, d is the communication distance (millimeter, mm) between a transmitter and a receiver, and N is a normally distributed variable with zero mean and standard deviation $\sigma_{N}$. Details about these parameters can be found in the literature [33]. Using PL(d), the signal to noise ratio (SNR) at a receiver can be expressed as follows:

(16) $$\;{\left(\mathrm{SNR}\right)}_{dB}=P_{r}-P_{n}\;$$

(17) $$\;P_{r}=P_{t}-PL\left(d\right)\;$$

(18) $$\;P_{n}=N_{thermal}+{\left(NF\right)}_{dB}+I_{dB}\;$$

where $P_{t}$ is transmission power and $N_{thermal}$ is thermal noise. The average path loss is shown in Figure 10. It is assumed that the channel condition does not change until the two-hop relay is completed or the two-hop relay fails beyond the maximum number of retransmissions.

In addition, each case of the proposed scheme in each hop is summarized in Table 5.

Then, energy efficiency η is derived from our previous work [22] as follows:

(19) $$\;\eta \equiv \frac{P_{succ}L_{info}}{E_{link,N1\to N2}+E_{link,N2\to H}}\;$$

(20) $$\;E_{link,\;\;A\to B}=\left(T_{TOT}+N_{tx}T_{ACK}\right)\left(P_{tx,RF}+P_{tx,circ}+P_{rx}\right)+N_{tx}\left(\varepsilon_{enc}+\varepsilon_{dec}\right)\;$$

(21) $$\;T_{TOT}=T_{SHR}+T_{PHR}+\overset{tr_{A\to B}}{\underset{i=1}{\sum }}\frac{L_{PSDU,i}}{R}\;$$

Here, $E_{link,\;A\to B}$ is the energy consumption of the communication link at each hop and $P_{succ}$ is the transmission success ratio, $T_{TOT}$ is the total duration of packet transmission, $T_{ACK}$ is the duration of ACK, $L_{PSDU,i}$ is the length of PSDU, $N_{tx}$ is the number of transmission, $P_{tx,RF}$ is the transmitter RF power consumption, $P_{tx,circ}$ is the transmitter circuitry power consumption, $P_{rx}$ is the receiver power consumption, and $\varepsilon_{enc}$ and $\varepsilon_{dec}$ are the encoding and decoding energies, respectively [34,35,36,37].

**Figure 11:**
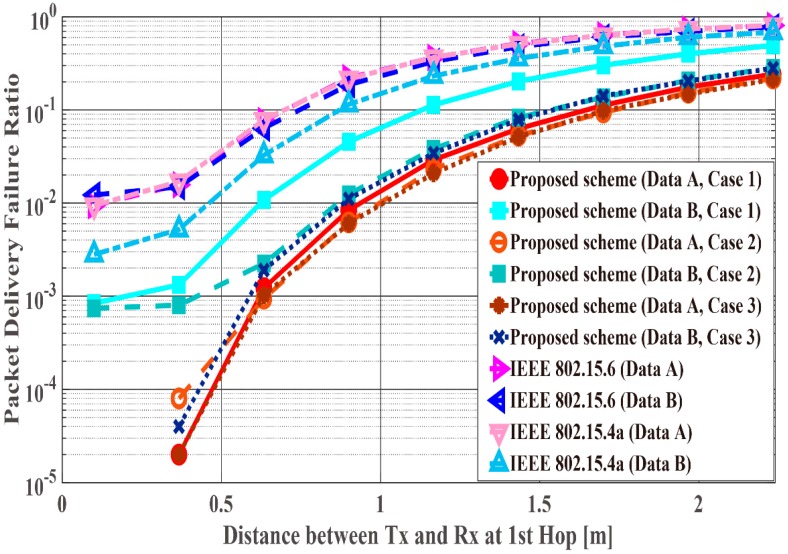
Packet delivery failure ratio with constant $d_{2nd}$.

**Figure 12:**
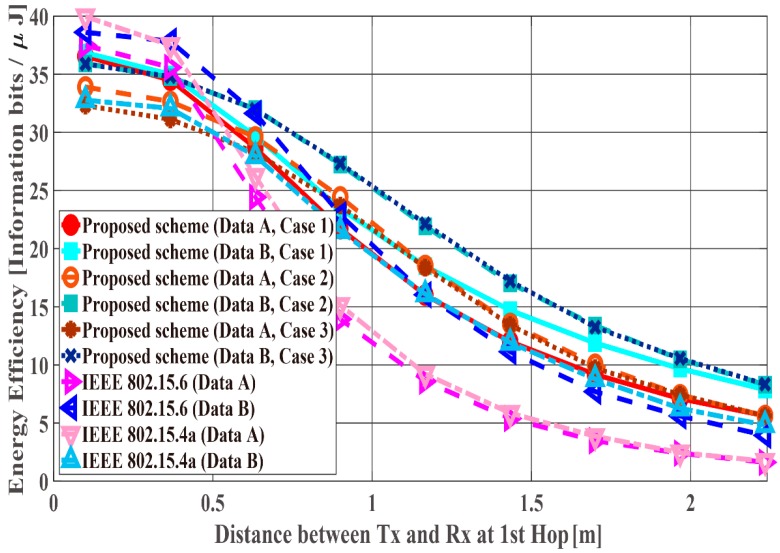
Energy efficiency with constant $d_{2nd}$.

**Figure 13:**
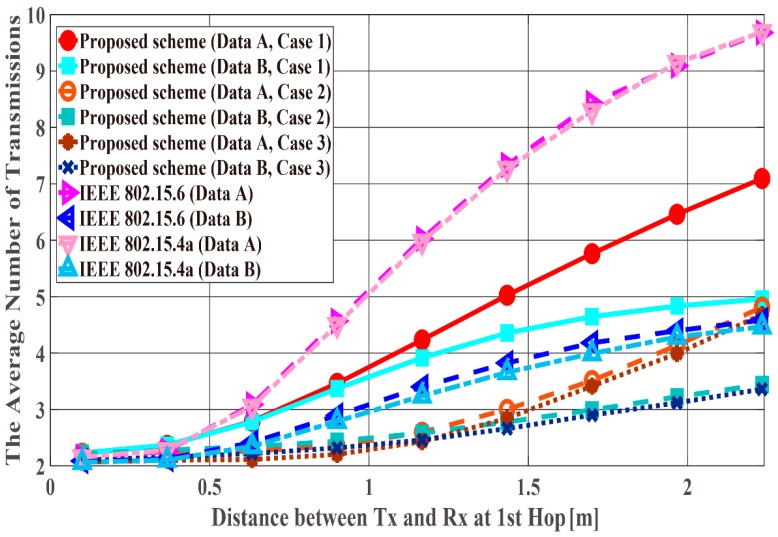
Average number of transmissions with constant $d_{2nd}$.

Figure 11, Figure 12 and Figure 13 show the performance results when the distance of the first hop $d_{1st}$ is changed from 10 centimeters (cm) to 3 m and the distance of the second hop $d_{2nd}$ is constant ($d_{2nd}$ = 40 cm). In this scenario, it can be said that the performance in the range in which the WBAN mainly operates (10 cm~1.5 m) and certain limitations of the WBAN system (1.5 m~2.3 m) are evaluated. PDFR means the ratio at which the two-hop relay failed beyond the maximum number of retransmissions. As can be seen, the proposed scheme satisfies the QoS requirements for data A and B as shown in Table 1, while IEEE Std. 802.15.6 and 15.4a do not. Hence, the proposed method can improve PER of Data A more, while it can improve the energy efficiency and the number of transmissions of Data B more. Conversely, Data B has better performances with respect to both standard schemes. The reason is that those standard schemes are not basically designed so that any QoSs can be satisfied. Hence, it can be considered that the performances of each mode of IEEE Std. 802.15.6 and error control schemes of IEEE Std. 802.15.4a were simply expressed. Also, that is one of problems of these standard schemes. Cases 2 and 3 show better energy efficiency and average number of transmissions than Case 1, because the coding rate of Case 2 and Case 3 is set appropriately for the channel SNR and the number of retransmissions is reduced by utilizing Scheme 2, while Case 1 uses only Scheme 1 and it requires a larger number of retransmissions. However, there is not a large difference between Cases 2 and 3 because $d_{2nd}$ is short and the error correcting capability of coding rate $r_{c}$ = 8/9 at the first transmission can reduce bit errors sufficiently. That is, there is no large difference between Schemes 1 and 2 with respect to the second hop.

**Figure 14:**
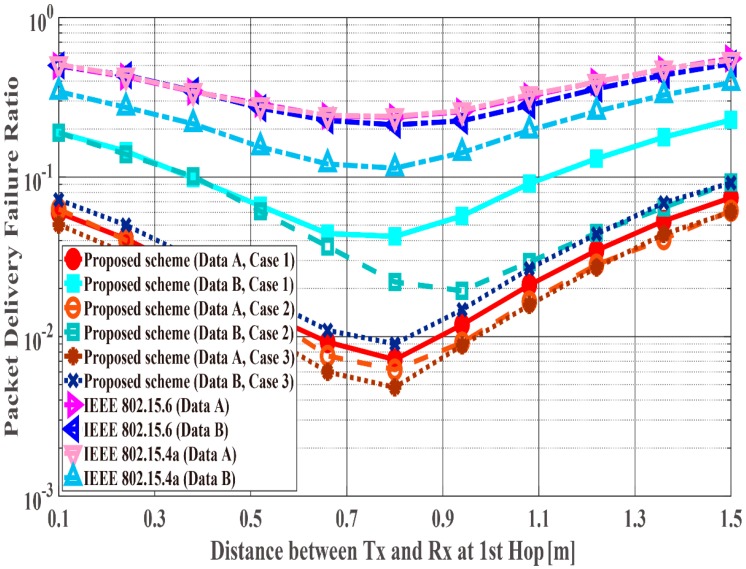
Packet delivery failure ratio with constant $d_{2hops}$.

**Figure 15:**
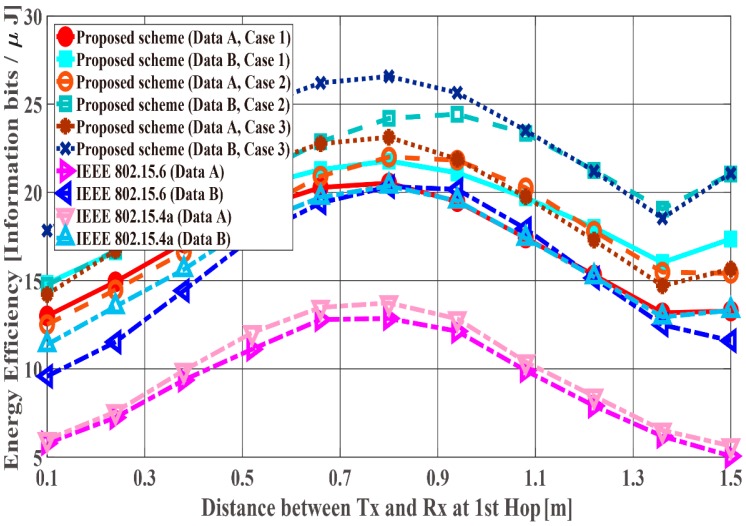
Energy efficiency with constant $d_{2hops}$.

**Figure 16:**
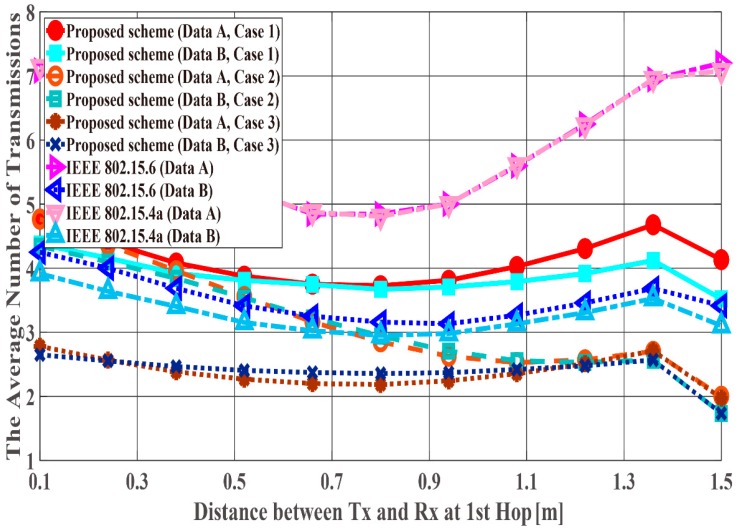
Average number of transmissions with constant $d_{2hops}$.

Figure 14, Figure 15 and Figure 16 show the performance results for fixed communication distance in two hops $d_{2hops}=d_{1st}$+$d_{2nd}$ (1.5 m) and varying the $d_{1st}$ and $d_{2nd}$ values. For $d_{1st}$ = 1.5 m, data are transmitted using only a single hop. Thus, the proposed scheme satisfies the QoS requirements for Data A and B, while both standard schemes approach do not, like in the first scenario. Also, when comparing the standard schemes and the proposed scheme, the performances of both standards are worse than the proposed one. For example, Data A of the proposed scheme satisfies PDFR <$\;10^{-2}$, while that of both standards do not satisfy PDFR <$\;10^{-1}$. This is because the correcting capability of error correcting codes used in those standards is lower than that of the proposed scheme. In other words, the standard schemes do not have sufficient correcting capability in a hop with poor channel conditions. Comparing Case 1 and Case 2, it is understood that Case 2 has better characteristics. The reason is that Case 2 can select a coding rate suitable for the channel condition by using Scheme 2 at the second hop. On the other hand, regarding Case 1, since Scheme 1 is used at both hops, it is considered that a hop having a bad channel condition is greatly affected. Then, Case 3 shows the best performance because Scheme 2 is used at both hops. In addition, all systems except Case 2 of the proposed scheme show the best performance when the communication distance of the first hop equals that of the second hop because $d_{1st}$ or $d_{2nd}$ becomes long (unlike the previous condition) and the long-distance communication influences performance in other cases.

### 6.2. Theoretical Analysis of Constant $d_{2hops}$

Here, we present a theoretical analysis when $d_{2hops}$ is fixed because this scenario appears to show the optimal point in Figure 14, Figure 15 and Figure 16. The reason for the optimized performances, except for Case 2, when $d_{1st}$ = $d_{2nd}$ = $d_{2hop}/2$ is described in this section.

The probability of transmission failure in the two-hop case $P_{fail,2hop}$ is expressed using the probability of transmission failure at each hop $P_{fail,1st}$, $P_{fail,2nd}$ as follows:

(22) $$\;P_{fail,2hop}=P_{fail,1st}+\;\left(1-P_{fail,1st}\right)P_{fail,2nd}\;$$

(23) $$\;P_{fail,1st}\left(d_{1st}\right)=\overset{\left\lceil \bar{tr_{N1\to N2}}-0.5\right\rceil }{\underset{i=1}{\prod }}P_{fail,i}\left(d_{1st}\right)\;$$

(24) $$\;P_{fail,2nd}\left(d_{1st}\right)=\overset{\left\lceil \bar{tr_{N2\to H}}-0.5\right\rceil }{\underset{i=1}{\prod }}P_{fail,i}\left(d_{2hop}-d_{1st}\right)\;$$

(25) $$\;0<P_{fail,1st}\left(d_{1st}\right),\;P_{fail,2nd}\left(d_{1st}\right)<1\;$$

(26) $$\;0<P_{fail,1st}{\left(d_{1st}\right)}^{\prime }\;$$

(27) $$\;P_{fail,2nd}{\left(d_{1st}\right)}^{\prime }<0\;$$

Here, $P_{fail,1st}{\left(d_{1st}\right)}^{\prime }$ = $\frac{dP_{fail,1st}\left(d_{1st}\right)}{d\left(d_{1st}\right)}$ and $P_{fail,2nd}{\left(d_{1st}\right)}^{\prime }$ = $\frac{dP_{fail,2nd}\left(d_{1st}\right)}{d\left(d_{1st}\right)}$. The communication distance in two hops $d_{2hops}$ is defined as follows:

(28) $$d_{2hops}=d_{1st}+d_{2nd}\;$$

(29) $$\;0<d_{1st},\;d_{2nd}<d_{2hop}\;$$

$P_{fail,2hop}$ is differentiated by $d_{1st}$ as follows:

(30) $$\;P_{fail,2hop}{}^{\prime }=P_{fail,1st}{}^{\prime }+P_{fail,1st}{}^{\prime }P_{fail,2nd}+\left(1-P_{fail,1st}\right)P_{fail,2nd}{}^{\prime }\;=\left(1-P_{fail,2nd}\right)P_{fail,1st}{}^{\prime }+\left(1-P_{fail,1st}\right)P_{fail,2nd}{}^{\prime }.$$

Here, the case that (30) = 0 is considered. Equation (30) is modified by the following equation:

(31) $$\;\frac{1}{1-P_{fail,1st}}P_{fail,1st}{}^{\prime }=-\frac{1}{1-P_{fail,2nd}}P_{fail,2nd}{}^{\prime }\;$$

Both sides of (31) are integrated by $d_{1st}$ as follows:

(32) $$\;-\ln\left(1-P_{fail,1st}\right)=\frac{1}{1-P_{fail,2nd}}\left(-\frac{dP_{fail,2nd}}{dd_{2nd}}\frac{dd_{2nd}}{dd_{1st}}\right)\;=-\ln\left(1-P_{fail,2nd}\right)+C.$$

Here, $d_{c}$ where $P_{fail,1st}\left(d_{c}\right)=P_{fail,2nd}\left(d_{c}\right)$ is considered. Under this condition, C=0. Thus, (32) is rewritten as follows:

(33) $$\;P_{fail,1st}-P_{fail,2nd}=0\;\;\overset{\left\lceil \bar{tr_{N1\to N2}}-0.5\right\rceil }{\underset{i=1}{\prod }}P_{fail,i}\left(d_{1st}\right)-\overset{\left\lceil \bar{tr_{N2\to H}}-0.5\right\rceil }{\underset{i=1}{\prod }}P_{fail,i}\left(d_{2hop}-d_{1st}\right)=0\;$$

$d_{1st},\;\bar{tr_{N1\to N2}},$ and $\bar{tr_{N2\to H}}$ that satisfies (33) are considered. Here, it is assumed that $d_{1st}=d_{2nd}=\frac{d_{2hop}}{2}$. Under this condition, (33) is satisfied when $\bar{tr_{N1\to N2}}=\bar{tr_{N2\to H}}$ in the computer simulations (except for Case 2) because, when $\bar{tr_{N1\to N2}}\neq \bar{tr_{N2\to H}}$, (27) is modified as follows:

(34) $$\;\overset{\left\lceil \bar{tr_{N2\to H}}-0.5\right\rceil }{\underset{i=1}{\prod }}P_{fail,i}\left(\frac{d_{2hop}}{2}\right)\left\{1-\overset{\left\lceil \bar{tr_{N1\to N2}}-0.5\right\rceil }{\underset{i=\left\lceil \bar{tr_{N2\to H}}-0.5\right\rceil +1}{\prod }}P_{fail,i}\left(\frac{d_{2hop}}{2}\right)\right\}=0\;$$

When (26) is satisfied,

(35) $$\;\overset{\left\lceil \bar{tr_{N2\to H}}-0.5\right\rceil }{\underset{i=1}{\prod }}P_{fail,i}\left(\frac{d_{2hop}}{2}\right)=0\;$$

or

(36) $$\;\overset{\left\lceil \bar{tr_{N1\to N2}}-0.5\right\rceil }{\underset{i=\left\lceil \bar{tr_{N2\to H}}-0.5\right\rceil +1}{\prod }}P_{fail,i}\left(\frac{d_{2hop}}{2}\right)=1\;$$

However, due to (27)–(29), (35) and (36) are not satisfied. Thus, it can be said that $\bar{tr_{N1\to N2}}=\bar{tr_{N2\to H}}$. On the other hand, for Case 2, (33) is not satisfied when $\bar{tr_{N1\to N2}}=\bar{tr_{N2\to H}}$ because $P_{fail,1st,i}\left(\frac{d_{2hop}}{2}\right)\neq P_{fail,2nd,i}\left(\frac{d_{2hop}}{2}\right)$. From Figure 14, Figure 15 and Figure 16, it can be observed that all cases (except Case 2) achieve optimal performance under the above conditions; thus, $d_{1st}=d_{2nd}=\frac{d_{2hop}}{2}$.

## 7. Conclusions

In this paper, the performance of our proposed QoS control scheme in the case of two-hop extension was evaluated. The PDFR, number of transmissions, and energy efficiency of our previously proposed system, IEEE Std. 802.15.6, and 15.4a were evaluated for this case. Also, two schemes (Schemes 1 and 2) were compared for the proposed method. The numerical results show that the proposed scheme outperforms the standard scheme in terms of the PDFR, number of transmissions, and energy efficiency. In addition, Case 3 (i.e., the coding rates change depending on the channel’s condition) showed better performance than the other cases at both hops. When $d_{2hops}$ was fixed, it was shown that performance became optimal when $d_{1st}=d_{2nd}$ (except Case 2) from computer simulations and theoretical analysis. This result is expected to greatly contribute to the optimization of how nodes and hubs are arranged when designing a WBAN.

In the future, an effective error control scheme for multi-hop WBANs should be considered. In addition, PHY evaluation indexes were mainly considered. Hence, evaluating the system delay and throughput in the network layer should be considered for multi-hop cases. As an extension of IEEE Std. 802.15.6, cases with greater than three hops should also be evaluated and analyzed theoretically.

## Figures and Tables

**Table 1:** QoS requirements of different data types.

Data Types	Data A	Data B
User priority	5	6
PER	$\leq 10^{-2}$	$\leq 10^{-1}$
Energy efficiency	low	high

**Table 2:** Maximum number of transmissions.

q	$q_{N1\to N2}$	$q_{N2\to H}$	$q_{max}$
Data A	11	$11-tr_{N1\to N2}$	11
Data B	5	5	10

**Table 3:** Main simulation parameters.

Parameter	Detail
Channel model	IEEE model CM3
Path loss model	IEEE model CM3
Bandwidth (*BW*)	499.2 MHz
Central frequency ($f_{c}$)	3993.6 MHz
Pulse shape	Gaussian mono pulse
Pulse duration ($T_{p}$)	2.003 ns
Modulation	DBPSK
FEC	$r_{c}=$ 8/9 to 1/16, K=3 Convolutional codes
Decoding	Soft decision Viterbi decoding
ARQ protocol	Weldon’s ARQ
Power spectral density ($P_{sd}$)	−41.3 dBm/MHz
Thermal noise density ($N_{0}$)	−174 dBm/Hz
Implementation losses (I)	3 dB
Receiver noise figure (*NF*)	5 dB
Tx RF power consumption ($P_{tx,RF}$)	37 μW
Tx circ. power consumption ($P_{tx,circ}$)	2 mW
Rx power consumption ($P_{rx}$)	20 mW
Number of pulses per bit ($N_{cpb}$)	2
Integer number of pulse waveform positions ($N_{w}$)	32
Uncoded data rate (*R*)	7.8 Mbps
Synch. header duration ($T_{SHR}$)	40.32 μs
PHY header durations ($T_{PHR}$)	82.052 μs
Information bit length ($L_{info}$)	306 bits
ACK length ($L_{ACK}$)	7 bytes

**Table 4:** Preset number of data copies in Weldon’s ARQ $n_{i}$.

i	1	2	3	4	5	6	7	8	9	10	11
Data A	1	4	4	5	5	6	6	7	7	8	8
Data B	1	1	2	3	4	-	-	-	-	-	-

**Table 5:** Cases for the proposed scheme of each hop.

	N1→N2	N2→H
Case 1	Scheme 1	Scheme 1
Case 2	Scheme 1	Scheme 2
Case 3	Scheme 2	Scheme 2
